# 2,5-Dibromo­terephthalic acid dihydrate

**DOI:** 10.1107/S1600536808027268

**Published:** 2008-08-30

**Authors:** Guang-Liang Song, Shan Liu, Hua-Jun Liu, Tao Zeng, Hong-Jun Zhu

**Affiliations:** a Department of Applied Chemistry, College of Science, Nanjing University of Technology, Nanjing 210009, People’s Republic of China

## Abstract

The asymmetric unit of the title compound, C_8_H_4_Br_2_O_4_·2H_2_O, contains one half-mol­ecule of 2,5-dibromo­terephthalic acid (DBTA) and one water mol­ecule. The DBTA mol­ecule is centrosymmetric. In the crystal structure, inter­molecular O—H⋯O hydrogen bonds link the mol­ecules, forming a three-dimensional framework.

## Related literature

For general background, see: Yao & Tour (1999 ▶). For a related structure, see: Singh & Bedi (1957 ▶).
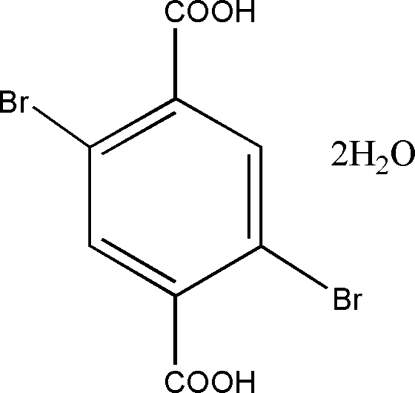


## Experimental

### 

#### Crystal data

C_8_H_4_Br_2_O_4_·2H_2_O
*M* *_r_* = 359.94
Monoclinic, 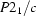

*a* = 10.670 (2) Å
*b* = 7.413 (1) Å
*c* = 7.074 (1) Å
β = 92.74 (3)°
*V* = 558.89 (15) Å^3^
*Z* = 2
Mo *K*α radiation
μ = 7.26 mm^−1^
*T* = 293 (2) K
0.10 × 0.10 × 0.08 mm

#### Data collection

Enraf–Nonius CAD-4 diffractometer
Absorption correction: ψ scan (North *et al.*, 1968 ▶) *T*_min_ = 0.530, *T*_max_ = 0.594 (expected range = 0.499–0.559)
1003 measured reflections
1003 independent reflections
763 reflections with *I* > 2σ(*I*)
3 standard reflections every 200 reflections intensity decay: none

#### Refinement

*R*[*F* ^2^ > 2σ(*F* ^2^)] = 0.048
*wR*(*F* ^2^) = 0.117
*S* = 1.05
1003 reflections
67 parameters
21 restraints
H-atom parameters constrained
Δρ_max_ = 0.55 e Å^−3^
Δρ_min_ = −0.70 e Å^−3^

### 

Data collection: *CAD-4 Software* (Enraf–Nonius, 1985 ▶); cell refinement: *CAD-4 Software*; data reduction: *XCAD4* (Harms & Wocadlo, 1995 ▶); program(s) used to solve structure: *SHELXS97* (Sheldrick, 2008 ▶); program(s) used to refine structure: *SHELXL97* (Sheldrick, 2008 ▶); molecular graphics: *SHELXTL* (Sheldrick, 2008 ▶); software used to prepare material for publication: *SHELXTL*.

## Supplementary Material

Crystal structure: contains datablocks I, global. DOI: 10.1107/S1600536808027268/ez2130sup1.cif

Structure factors: contains datablocks I. DOI: 10.1107/S1600536808027268/ez2130Isup2.hkl

Additional supplementary materials:  crystallographic information; 3D view; checkCIF report

## Figures and Tables

**Table 1:** Hydrogen-bond geometry (Å, °)

*D*—H⋯*A*	*D*—H	H⋯*A*	*D*⋯*A*	*D*—H⋯*A*
O*W*—H*WA*⋯O1^i^	0.85	2.11	2.903 (9)	155
O*W*—H*WB*⋯O1^ii^	0.85	2.22	2.944 (9)	142
O2—H2*A*⋯O*W*	0.82	1.75	2.566 (8)	177

Symmetry codes: (i) 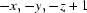; (ii) 
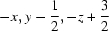
.

**Fig. 1.**
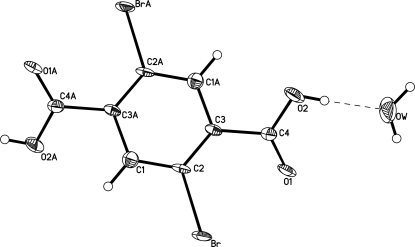
The molecular structure of (I), showing the atom labelling scheme. Anisotropic displacement parameters are shown at the 50% probability level.

## supplementary crystallographic information

### Comment

2,5-Dibromoterephthalic acid (DBTA) is an important intermediate in the preparation of flame-retardant polymers (Yao *et al.*, 1999). We report herein the crystal structure of the title compound (I).

The asymmetric unit of I (Fig. 1), contains one half of a molecule of 2,5-dibromoterephthalic acid (DBTA), which is related to the other half by a center of symmetry, and one water molecule. Three neighbouring DBTA molecules are linked through one water molecule by intermolecular O—H···O hydrogen bonds, to form a three dimensional framework.

### Experimental

The title compound was prepared according to the method described by Singh & Bedi (1957). Crystals of (I) suitable for X-ray analysis were obtained by dissolving DBTA (2.0 g) in water (80 ml) and evaporating slowly at room temperature for about 15 d.

### Refinement

Anisotropic parameters of the C atoms in the phenyl ring were restrained to have equal components and approximately isotropic behavior. H atoms were positioned geometrically, with O—H = 0.82 (for OH) and 0.85 (for H_2_O) and C—H = 0.93 and 0.96 Å for aromatic and methyl H, respectively, and constrained to ride on their parent atoms, with *U*_iso_(H) = *xU*_eq_(C/O), where *x* = 1.2 for aromatic H and *x* = 1.5 for other H.

### Crystal data

**Table:**

C_8_H_4_Br_2_O_4_·2H_2_O	*F*_000_ = 348
*M**_r_* = 359.94	*D*_x_ = 2.139 Mg m^−^^3^
Monoclinic, *P*2_1_/*c*	Mo *K*α radiation λ = 0.71073 Å
*a* = 10.670 (2) Å	Cell parameters from 25 reflections
*b* = 7.413 (1) Å	θ = 10–13º
*c* = 7.074 (1) Å	µ = 7.26 mm^−^^1^
β = 92.74 (3)º	*T* = 293 (2) K
*V* = 558.89 (15) Å^3^	Block, colorless
*Z* = 2	0.10 × 0.10 × 0.08 mm

### Data collection

**Table:**

Enraf–Nonius CAD-4 diffractometer	*R*_int_ = 0.0000
Radiation source: fine-focus sealed tube	θ_max_ = 25.2º
Monochromator: graphite	θ_min_ = 1.9º
*T* = 293(2) K	*h* = −12→12
ω/2θ scans	*k* = 0→8
Absorption correction: ψ scan(North *et al.*, 1968)	*l* = 0→8
*T*_min_ = 0.530, *T*_max_ = 0.594	3 standard reflections
1003 measured reflections	every 200 reflections
1003 independent reflections	intensity decay: none
763 reflections with *I* > 2σ(*I*)	

### Refinement

**Table:**

Refinement on *F*^2^	Secondary atom site location: difference Fourier map
Least-squares matrix: full	Hydrogen site location: inferred from neighbouring sites
*R*[*F*^2^ > 2σ(*F*^2^)] = 0.048	H-atom parameters constrained
*wR*(*F*^2^) = 0.117	*w* = 1/[σ^2^(*F*_o_^2^) + (0.06*P*)^2^ + 1.5*P*] where *P* = (*F*_o_^2^ + 2*F*_c_^2^)/3
*S* = 1.05	(Δ/σ)_max_ < 0.001
1003 reflections	Δρ_max_ = 0.55 e Å^−^^3^
67 parameters	Δρ_min_ = −0.70 e Å^−^^3^
21 restraints	Extinction correction: none
Primary atom site location: structure-invariant direct methods	

### Special details

**Table:**

Geometry. All e.s.d.'s (except the e.s.d. in the dihedral angle between two l.s. planes) are estimated using the full covariance matrix. The cell e.s.d.'s are taken into account individually in the estimation of e.s.d.'s in distances, angles and torsion angles; correlations between e.s.d.'s in cell parameters are only used when they are defined by crystal symmetry. An approximate (isotropic) treatment of cell e.s.d.'s is used for estimating e.s.d.'s involving l.s. planes.
Refinement. Refinement of *F*^2^ against ALL reflections. The weighted *R*-factor *wR* and goodness of fit *S* are based on *F*^2^, conventional *R*-factors *R* are based on *F*, with *F* set to zero for negative *F*^2^. The threshold expression of *F*^2^ > σ(*F*^2^) is used only for calculating *R*-factors(gt) *etc*. and is not relevant to the choice of reflections for refinement. *R*-factors based on *F*^2^ are statistically about twice as large as those based on *F*, and *R*- factors based on ALL data will be even larger.

### Fractional atomic coordinates and isotropic or equivalent isotropic displacement parameters (Å^2^)

**Table:**

	*x*	*y*	*z*	*U*_iso_*/*U*_eq_	
Br	0.31754 (7)	0.35260 (9)	0.52687 (10)	0.0366 (3)	
OW	−0.0069 (5)	−0.2828 (11)	0.7105 (11)	0.093 (3)	
HWA	−0.0608	−0.2028	0.6780	0.111*	
HWB	−0.0299	−0.3786	0.7651	0.111*	
O1	0.1532 (5)	0.0220 (6)	0.5124 (8)	0.0495 (14)	
O2	0.2259 (5)	−0.2252 (7)	0.6570 (9)	0.0554 (15)	
H2A	0.1515	−0.2402	0.6767	0.083*	
C1	0.5431 (6)	0.1725 (10)	0.4729 (9)	0.034	
H1A	0.5725	0.2885	0.4518	0.040*	
C2	0.4182 (6)	0.1457 (8)	0.5085 (9)	0.0276 (13)	
C3	0.3736 (6)	−0.0245 (8)	0.5317 (8)	0.0255 (13)	
C4	0.2396 (6)	−0.0748 (9)	0.5662 (9)	0.0314 (15)	

### Atomic displacement parameters (Å^2^)

**Table:**

	*U*^11^	*U*^22^	*U*^33^	*U*^12^	*U*^13^	*U*^23^
Br	0.0487 (4)	0.0111 (4)	0.0496 (5)	0.0050 (3)	−0.0007 (3)	0.0002 (3)
OW	0.030 (3)	0.111 (6)	0.139 (7)	0.008 (3)	0.013 (3)	0.073 (5)
O1	0.045 (3)	0.016 (3)	0.087 (4)	−0.004 (2)	−0.002 (3)	0.015 (3)
O2	0.051 (3)	0.028 (3)	0.087 (4)	−0.006 (3)	−0.004 (3)	0.029 (3)
C1	0.034	0.034	0.034	0.000	0.002	0.000
C2	0.043 (3)	0.009 (3)	0.030 (3)	0.002 (3)	−0.009 (3)	0.000 (3)
C3	0.038 (3)	0.013 (3)	0.025 (3)	−0.002 (3)	−0.003 (2)	−0.004 (3)
C4	0.035 (3)	0.022 (3)	0.038 (4)	0.003 (3)	0.004 (3)	0.005 (3)

### Geometric parameters (Å, °)

**Table:**

Br—C2	1.880 (6)	C1—C2	1.383 (8)
OW—HWA	0.8500	C1—C3^i^	1.413 (9)
OW—HWB	0.8500	C1—H1A	0.9300
O1—C4	1.215 (8)	C2—C3	1.361 (8)
O2—C4	1.299 (8)	C3—C1^i^	1.413 (9)
O2—H2A	0.8200	C3—C4	1.508 (9)
			
HWA—OW—HWB	120.0	C1—C2—Br	117.0 (5)
C4—O2—H2A	109.5	C2—C3—C1^i^	119.5 (6)
C2—C1—C3^i^	120.4 (6)	C2—C3—C4	126.0 (6)
C2—C1—H1A	119.8	C1^i^—C3—C4	114.5 (5)
C3^i^—C1—H1A	119.8	O1—C4—O2	124.1 (6)
C3—C2—C1	120.1 (6)	O1—C4—C3	121.0 (6)
C3—C2—Br	122.9 (5)	O2—C4—C3	115.0 (6)
			
C3^i^—C1—C2—C3	−2.7 (10)	Br—C2—C3—C4	2.8 (9)
C3^i^—C1—C2—Br	176.2 (5)	C2—C3—C4—O1	26.1 (10)
C1—C2—C3—C1^i^	2.7 (10)	C1^i^—C3—C4—O1	−154.9 (6)
Br—C2—C3—C1^i^	−176.2 (5)	C2—C3—C4—O2	−153.4 (7)
C1—C2—C3—C4	−178.3 (6)	C1^i^—C3—C4—O2	25.6 (8)

Symmetry codes: (i) −*x*+1, −*y*, −*z*+1.

### Hydrogen-bond geometry (Å, °)

**Table:**

*D*—H···*A*	*D*—H	H···*A*	*D*···*A*	*D*—H···*A*
OW—HWA···O1^ii^	0.85	2.11	2.903 (9)	155
OW—HWB···O1^iii^	0.85	2.22	2.944 (9)	142
O2—H2A···OW	0.82	1.75	2.566 (8)	177

Symmetry codes: (ii) −*x*, −*y*, −*z*+1; (iii) −*x*, *y*−1/2, −*z*+3/2.
